# Supplementary material for: Chromothripsis during telomere crisis is independent of NHEJ, and consistent with a replicative origin
Source: Genome Res. 2019 May;29(5):737–49. doi: 10.1101/gr.240705.118 (PMC6499312; doi:10.1101/gr.240705.118)
Supplement: Supplemental Material [file supp_gr.240705.118_Supplemental_file_1.zip › contigs/annotated_contigs/DB48/contig.2.DB48_length_721_mean_cov_8.23023578363.docx]

**DB48_length_721_mean_cov_8.23023578363**

```
CAGCTTTTCTTAAAGGTAGTTAGCCATGTCCATATAGTTAAGAGTTGTGCTGTAATTCTGCTGTGAGATTAAAAATCAGGTATATGATT
 >chr1:107560595-107561023 + E=9e-246
TAAAATATATAATATTCTCACTATTTAGTAACTAGAAAAGTCAGGGTCATAATTGGTGTGACCAAATTTTCTGACACTAGATTGGGAGA

GTTCTGAGACTTTTTACTGCTTACACGTCTAGGTCCACCAGGAATAGTCCAAGAACACATACCGGCTCCAAATGATTGCAACTTTATTT

CACAAATGCTCACAAACTATTATTTCATAATTGATTTTAGAATCCTGCCTAGATCTATTGAGGGAACTACAGTTTATAAATAAAATATC

TTGATACTTACTGAAAATGTAGATCTTATCTATCCTTACAAATCTTTCATGATTCTTTCAACATTTCCTACA|GCTCAGGT|GAGGCAG
                                                                                 >chr1:
CTCACCTGAGTTTCAATGTCCAGAGTTTTTATTGAGGCTTTATTACATAGGTGTGATTGATTGATTTCCCACAAGATTGTACTCAACCT
107564232-107564517 + E=1e-156
CTAGGATGACCGATAGATACCATACAACCCAACCCCCCTACTCTAAATCACATGGTTGCTATTTATGCTATTTATGGCATGAACAGCGC

CATTCTAAGGCTATTGGGTATGGATGGCATCGCTAATAAGATCTAGTTTAACTGGCACCAGTCAATGTATGTCATACCTGAGAGGAGTG

TCTTTGTTTAC
```
